# The Clinical Research Association

**Published:** 1897-04-10

**Authors:** 


### THE CLINICAL RESEARCH ASSOCIATION.

The report and handbook of the Clinical Research Association just issued is not only a record of very good work, but contains much useful information as to the best way of packing and forwarding specimens for examination. That the association fills a want is shown by the large number of specimens, of one sort and another, which have been sent up for examination, amounting to no less than 7,227 in the course of the year. To the very busy man it must be a matter of extreme convenience to have at his disposal, at certain fixed fees, the services of experts in the examination of morbid specimens; while to those who are willing and prepared to do the mass of their pathological work themselves, it must no less be an advantage to be able, in case of doubt, to obtain the opinion of men whose experience in the examination of such specimens must necessarily be very large. The laboratories and offices are situated at 1, Southwark Street, S.E.

